# Effect of Inulin on Rheological Properties and Emulsion Stability of a Reduced-Fat Salad Dressing

**DOI:** 10.1155/2024/4229514

**Published:** 2024-07-09

**Authors:** Sornchai Sinsuwan

**Affiliations:** School of Human Ecology (Program in Food Nutrition and Applications) Sukhothai Thammathirat Open University, Nonthaburi 11120, Thailand

## Abstract

This study is aimed at investigating the potential use of inulin in a reduced-fat salad dressing to improve its rheological properties, fat globule size distribution, and emulsion stability. The reduced-fat salad dressing, which has 50% less fat compared to the full-fat counterpart (control), was prepared with varying inulin concentrations (10, 12.5, 15, 17.5, and 20% *w*/*w*). The full-fat and reduced-fat salad dressings exhibited a non-Newtonian shear-thinning behavior. Power law model was used to describe the rheological properties. Results showed that the flow behavior index (*n*) and consistency coefficient (*K*) were greatly affected by the concentration of inulin. A greater pseudoplasticity and apparent viscosity of the reduced-fat samples were achieved with a higher concentration of inulin. Oscillatory tests showed that the storage modulus (*G*′) and loss modulus (*G*^″^) values increased with increasing inulin concentration. All samples displayed characteristics of a viscoelastic solid, as evidenced by a greater *G*′ than *G*^″^. Regarding the size distribution of the oil droplets, the reduced-fat salad dressing containing a higher inulin content was observed to have a larger droplet size. All reduced-fat samples, similar to the full-fat counterparts, exhibited stability with no cream separation over one month of storage at 4°C, as determined by visual observation. Additionally, the reduced-fat salad dressings supplemented with 17.5 and 20% inulin exhibited stability against cream separation, comparable to the full-fat counterpart (*p* > 0.05), as measured by the thermal stress test (80°C for 30 min) with centrifugation. The sensory acceptance scores for reduced-fat salad dressing with 15 and 17.5% inulin, ranging from approximately 6.28 to 7.63 on a 9-point hedonic scale for all evaluated attributes (appearance, color, aroma, texture, taste, and overall acceptability), were not significantly different from those of the full-fat counterpart (*p* > 0.05). This study demonstrated that inulin may be a suitable ingredient in reduced-fat salad dressings.

## 1. Introduction

Salad dressing is one of the most preferred food items, consisting of vegetable oil, egg yolks, lemon juice or vinegar, and seasonings [1]. The salad and mayonnaise dressing markets are experiencing rapid growth, with a compound annual growth rate (CAGR) of 6.1% from 2022 to 2028. This growth can be attributed to the expanding variety of dressings available in markets around the world [2]. Importantly, there is a growing trend towards health consciousness and awareness of the risks of excess fat consumption, as it causes cardiovascular disease, cognitive dysfunction, diabetes, and obesity. Consumers' interest in low-fat foods has increased. Nowadays, research is exploring ways to make low-fat and low-calorie foods without losing quality. Consequently, several fat replacers are now used in salad dressing formulations to reduce the negative effects on the finished product when fat is reduced or removed.

A fat replacer is a substance that mimics the properties of fat in food while being lower in calories. The fat replacer is usually categorized into three classes based on its composition: lipids, proteins, and carbohydrate-based products. Carbohydrate-based fat replacers are widely used to stabilize oil-in-water emulsions and control their rheological properties [3, 4]. Inulin is a soluble dietary fiber that is polymerized into 2–60 monomers of fructose [5]. Since it is not hydrolyzed by human digestive enzymes, it is able to pass through the large intestine intact. Importantly, inulin is prebiotic and increases the growth of beneficial microbes in the intestine that compete with pathogenic bacteria. Inulin can be employed as a low-calorie sweetener, fat replacer, gelling agent, viscosity modifier, texture modifier, nondigestible fiber, and prebiotic in various foods. There are numerous applications for inulin in foods, including minced meats [6], chicken sausages [7], mashed potatoes [8], chocolate milk [9], yogurt [10], surimi [11], whey protein isolates [12], ice cream [13], extruded flour products [14], margarine [15], and cheese [16]. In addition, it has been reported that inulin can be used as a fat replacer in food emulsions in several studies. A low-fat mayonnaise containing 10% long-chain inulin (23 units) displayed a viscoelastic behavior comparable to that of a mayonnaise containing 3% modified starch [17]. A low-fat mayonnaise made from 5% long-chain inulin (22–25 units), 5% short-chain inulin (2–7 units), and 1.5% acetylated potato distarch phosphate (modified starch) showed similar physical, rheological, and organoleptic properties to commercial mayonnaise [18]. Therefore, inulin may have potential applications in salad dressings. Furthermore, the use of inulin as a fat replacer has attracted consumer interest due to its potential to reduce the caloric content of salad dressings and function as a prebiotic oligosaccharide and soluble dietary fiber.

Salad dressing is a popular condiment around the world. It is commonly used as a dressing for salads, as a sandwich spread, or as a dipping sauce. Salad dressing is similar to mayonnaise but usually has a thinner consistency and a slightly sweeter taste. Generally, salad dressing contains about 50% fat, resulting in approximately 68 calories from fat in one tablespoon (15 grams). Moreover, fat plays an important role in the rheological properties and sensory characteristics of salad dressing as one of its primary ingredients. There is an interest in the production of reduced-fat salad dressings and in the substitution of fat without altering the product consistency. Recently, there has been a report that the use of inulin (17.5%) as a fat replacer in salad dressing has reduced 50% of the fat without detriment to quality [19]. A 30% reduction in the energy density of the reduced-fat salad dressing containing 17.5% inulin (4.3 kcal/g) compared to the full-fat salad dressing (6.1 kcal/g) was also observed [19]. According to the results of this previous study, it was possible to obtain well-accepted salad dressings with a 50% reduction in fat content. So far, there is little information on the effects of inulin on the rheological properties and stability of reduced-fat salad dressings. Therefore, the purpose of this study was to study the rheological properties, fat globule size distribution, and emulsion stability of reduced-fat salad dressings at various concentrations of inulin.

## 2. Materials and Methods

### 2.1. Materials

Native inulin (Frutafit® IQ) was purchased from Sensus, Roosendaal, Netherlands. All other ingredients used in the preparation of salad dressing samples, such as soybean oil, egg yolk, sugar, vinegar, and salt, were purchased from a local market in Nakhon Ratchasima, Thailand.

### 2.2. Salad Dressing Preparation

Salad dressing was prepared according to the procedure described in previous works [19]. A preliminary experiment was conducted with varying concentrations of inulin (2-20%, *w*/*w*) to determine the viscosity of reduced-fat salad dressings. Based on visual observations of the dressings pouring onto a watch glass, a concentration of 10–20% showed the desired viscosity compared to full-fat salad dressing and was selected for the experiment. The receipts of full-fat (FF) salad dressing as a control and reduced-fat (RF) counterparts are shown in Table 1. Five hundred grams of each sample of salad dressing was prepared for this study. Briefly, vinegar, sugar, salt, and water were mixed, followed by the addition of egg yolk. This mixture was blended at a speed of 4,500 rpm for 45 seconds (HR 2117, Philips, Thailand). Soybean oil was added slowly to the mixture while blending at a speed of 5,300 rpm for 60 seconds. The salad dressing samples were transferred to glass bottles with screw caps and stored at 4°C until further analysis.

The reduced-fat salad dressings were prepared with the above-mentioned procedure, except the inulin was mixed with dried ingredients (sugar and salt) before blending with water and vinegar.

### 2.3. Steady Flow Behavior

The steady flow behavior of full-fat and reduced-fat salad dressings was measured using a rheometer (MCR 52, Anton Paar GmbH, Austria) with a cone-plate geometry (2° cone angle, 50 mm diameter) with a gap size of 0.205 mm. All samples were measured at 25°C. Flow characteristics were analyzed by using the power law model as follows:

(1) $$\begin{array}{c} \tau =K{\dot{\gamma }}^{n}, \end{array}$$

where *τ* is the shear stress (Pa), $\dot{\gamma }$ is the shear rate (s^−1^), *K* is the consistency coefficient (Pa·s^n^), and *n* is the flow behavior index (-).

### 2.4. Viscoelastic Behavior

The viscoelastic behavior of the full-fat and reduced-fat salad dressings was analyzed using the rheometer (MCR 52, Anton Paar GmbH, Austria) with a cone-plate geometry (2° cone angle, 50 mm diameter). The measurement was conducted with a gap size of 0.205 mm and at a temperature of 25°C. The linear viscoelastic range was determined with a strain sweep (strain range of 0.01-1,000%) at a fixed frequency of 1 Hz. Subsequently, a dynamic frequency sweep was conducted by applying a constant strain of 1%, which was within the linear region, over an angular frequency range of 0.1–100 rad/s. The storage modulus (*G*′) and loss modulus (*G*^″^) were recorded.

### 2.5. Particle Size Analysis

The particle size distribution of the sample was measured at 25°C using deionized water as the solvent with a laser scattering particle size distribution analyzer LA-950V2 (Horiba Instruments, Inc., CA, USA). The particle size distribution curve, the surface-volume mean diameter *D*[3, 2], and the weight-average diameter *D*[4, 3] were reported. The *D*[3, 2] and *D*[4, 3] were calculated using the following equation:

(2) $$\begin{array}{c} D\left[3,2\right]=\frac{\sum n_{i}d_{i}^{3}}{\sum n_{i}d_{i}^{2}},\\ D\left[4,3\right]=\frac{\sum n_{i}d_{i}^{4}}{\sum n_{i}d_{i}^{3}}, \end{array}$$

where *n*_*i*_ is the number of particle diameter *d*_*i*_. Each sample was measured in triplicate, and the data were presented as average.

### 2.6. Optical Microscopy

The microstructure of the prepared emulsion samples was observed using an optical microscope (Eclipse Ti-E, Nikon, Japan). A microscope glass slide was covered with a droplet of each sample and observed at a magnification of 400x.

### 2.7. Emulsion Stability

The emulsion stability under thermal stress of the full-fat and reduced-fat salad dressings was measured according to the method of Román et al. [20], with some modifications. Samples of 10 g (*F*_0_) were placed in 50 mL centrifuge tubes and heated in a water bath at 80°C for 30 min to promote destabilization. Subsequently, the heated samples were centrifuged at 4,000 × *g* (Sorvall Legend MACH 1.6R Centrifuge, Thermo Electron LED Gmbh, Osterode, Germany) for 15 min at room temperature. The separated layer was removed and weighed (*F*_1_). The stability of the emulsion was calculated using the following equation:

(3) $$\begin{array}{c} \text{Stability}=100-\left(\frac{F_{1}}{F_{0}}\times 100\right). \end{array}$$

The emulsion stability of both full-fat and reduced-fat salad dressings was evaluated during storage at 4°C. Each 25 g sample of salad dressing (*F*_0_) was placed in 50 mL centrifuge tubes and stored at 4°C. The creaming of the emulsion was monitored by visual observation and the centrifugation method at 0, 3, 6, 9, 12, 15, 20, 25, and 30 days of storage. Centrifugation method was determined by centrifugation at 4,000 × *g* (Sorvall Legend MACH 1.6R Centrifuge, Thermo Electron LED Gmbh, Osterode, Germany) for 15 min at room temperature. The visible serum separation layer was weighed (*F*_1_). The emulsion stability was calculated as described above.

### 2.8. Sensory Analysis

The sensory analysis was performed under controlled temperature and lighting conditions. Fifty untrained panelists evaluated the sensory characteristics (appearance, color, texture, aroma, taste, and overall acceptability) of the full-fat and reduced-fat salad dressings using a 9-point structured hedonic scale, where 1 represented “dislike extremely,” 5 represented “neither like nor dislike,” and 9 represented “like extremely.” Each panelist received six coded samples presented in disposable white cups. The codes were three-digit random numbers. The samples were presented to the panelists on a tray in individual booths and served one at a time at room temperature. Orders of serving were completely randomized. The panelists were asked to drink water between the samples to avoid aftertaste.

### 2.9. Statistical Analysis

At least two replicates were run for each analysis, except sensory evaluation, which was performed with two replications. Randomized complete block design (RCBD) was used for sensory analysis to block error from between and within treatment and panelists. The one-way analysis of variance (ANOVA) and Duncan multiple range test (DMRT) were used to analyze the significance of the difference in the rheological parameter, particle size, sensory analysis, and emulsion stability of the salad dressing. All statistical analyses were determined at 5% level of probability (*p* ≤ 0.05). Data are presented as the mean and standard deviation. The result was performed using the SPSS version 24.0 Statistical Software Package (SPSS, Inc., Chicago, IL, USA).

## 3. Results and Discussion

### 3.1. Steady Flow Behavior

**Figure 1:**
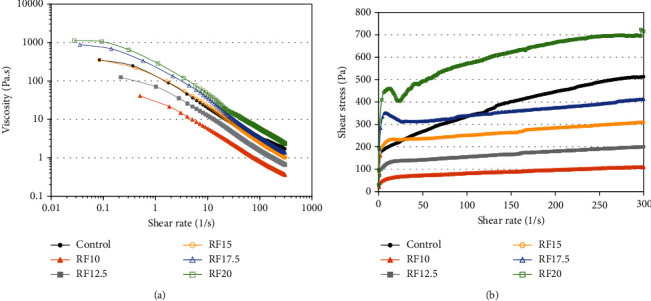
(a) Apparent viscosity and (b) shear stress versus shear rate for the full-fat salad dressing (control) and the reduced-fat salad dressing supplemented with inulin at 10 (RF10), 12.5 (RF12.5), 15 (RF15), 17.5 (RF17.5), and 20% (RF20).

A nonlinear, gradual decrease in apparent viscosity (Figure 1(a)) and an increase in shear stress (Figure 1(b)) were observed in both the full-fat and reduced-fat salad dressings with an increasing shear rate. This flow pattern is a typical rheological property of emulsions that resemble salad dressings. This was generally in good accordance with previous findings regarding the flow behavior of Italian salad dressing [21], low-fat soy-based salad dressing [22, 23], low-fat emulsion prepared with pregelatinized potato starch [24], pectin [25], inulin and modified starch [18], lentil flours [26], xanthan gum and tomato seed oil by-products [27], and modified starch and rice bran [28]. When the shear rate increased, the oil droplets in the full-fat emulsion, which is dispersed in a continuous phase with egg yolks as an emulsifier, could be disrupted. This could cause an aggregation of oil droplets to become deformed, leading to a gradual decrease in flow resistance (Figures 1(a) and 1(b)). In reduced-fat salad dressing, the weak intermolecular bonds between inulin molecules and other components in the continuous phase may be disrupted, and these molecules could be reoriented, leading to a decrease in apparent viscosity as the shear rate increases (Figures 1(a) and 1(b)). Hence, all samples exhibited non-Newtonian shear-thinning (pseudoplastic) behavior. This flow behavior is desirable for salad dressing as it allows for convenient pouring and spreading of salads.

The apparent viscosity of the reduced-fat salad dressing increased with increasing inulin concentration at the same point of each shear rate (Figure 1(a)). This result corresponded to the apparent viscosity obtained from the Brookfield viscometer in the previous report [19]. In addition, the higher shear stress was observed as an increase in inulin concentration (Figure 1(b)). Previous studies have also indicated that the viscosity and shear stress of low-fat emulsions were significantly influenced by hydrocolloid concentrations, as reported in pregelatinized potato starch [24], 4*α*GTase-modified rice starch [29], guar gum [30], and *β*-glucan [31]. The native inulin used in this study consists of both linear oligofructose and polyfructose [32]. It contained a wide range of degrees of polymerization, from 2 to 60, with an average chain length of 8 to 13 monomers, as reported by the manufacturer's specifications. Typically, long-chain inulin, often with a degree of polymerization (DP) greater than 10, has a greater impact on viscosity [33]. Longer-chain saccharides exhibit gel-forming ability at lower concentrations, while short-chain saccharides do not form gels [34]. Our results revealed that reduced-fat salad dressing supplemented with native inulin at 15 and 17.5% exhibited similar apparent viscosity to full-fat salad dressing at high shear rates (Figure 1(a)), indicating that these samples performed with flow consistency in applications where high shear rates were encountered as the same as full-fat salad dressing. When inulin is thoroughly mixed with an aqueous liquid, it is capable of developing a particulate gel that enhances the viscosity, stability, and texture of the gel system [35].

To assess the flow behavior characteristics, the steady shear rheological analysis data of all samples was applied to the power law model (Table 2). The *R*^2^ values of all samples were greater than 0.85, implying that the power law model provided an acceptable fit for the experimental data. All samples exhibited shear-thinning behavior, because their flow behavior index (*n*) was less than one. Typically, salad dressing is desired with an *n* value close to zero [27]. The *n* values of the reduced-fat salad dressing ranged from 0.093 to 0.190, while those of the full-fat counterpart were 0.303. The reduced-fat salad dressing supplemented with inulin showed a very low *n* value, suggesting a pronounced pseudoplasticity. Based on these results, the reduced-fat salad dressing would be easier to apply or pour onto meals because it flows more easily. In addition, an interesting finding revealed that when the inulin concentration reached 17.5 and 20%, the flow curve suddenly changed shape, in which the line dipped slightly and then continued upward at a low shear rate (Figure 1(b)). It has been reported that inulin gels were formed at 15% and high shear rate [33]. Inulin molecules could be fully stretched in the water at a high concentration (≥17.5%), and the high shear rate during emulsion preparation increased the probability of the molecules forming associations, leading to gel-like formation. When the shear rate reached a critical value, molecular interactions were disrupted, resulting in the deformation of the inulin gel.

The consistency index (*K*) of the reduced-fat salad dressing increased from 35.03 to 257.31 Pa·s^n^ when the amount of inulin added increased from 10 to 20%. In comparison, the consistency index of the full-fat dressing was 87.77 Pa·s^n^ (Table 2). In general, *K* is used to describe the resistance to flow or deformation of materials. This result indicated that the *K* value depends on the amount of vegetable oil and inulin. The reduced-fat dressing containing 12.5% inulin has nearly the same *K* value as the control, which contained 50% more fat content (Table 2). Our findings demonstrated that the addition of inulin as a flow behavior characteristic can compensate for the decrease in oil content. It might explain that the inulin molecule is a hydrophilic polysaccharide that can absorb and retain water. The individual hydrated inulin molecules and the entanglements between inulin chains cause an increase in overall bulkiness and flow resistance. Thus, a higher inulin concentration in the salad dressing would be more effective in reducing the mobility of the continuous phase and promoting a stronger structure of the salad dressing, leading to higher viscosity and greater consistency as the *K* value increased.

### 3.2. Viscoelastic Behavior

**Figure 2:**
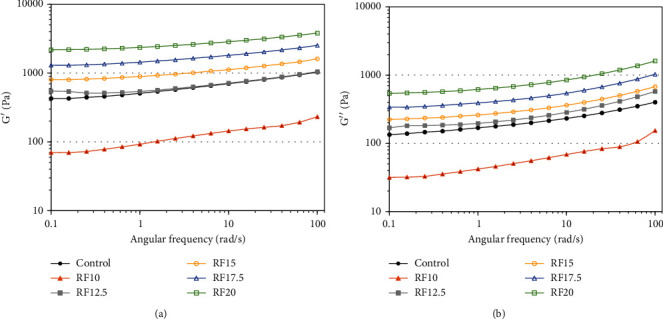
(a) Storage modulus (*G*′) and (b) loss modulus (*G*^″^) rheograms versus angular frequency for the full-fat salad dressing (control) and the reduced-fat salad dressing supplemented with inulin at 10 (RF10), 12.5 (RF12.5), 15 (RF15), 17.5 (RF17.5), and 20% (RF20).

The dynamic rheological behavior of salad dressing is shown in Figures 2(a) and 2(b). The storage modulus (*G*′) values of all samples were greater than the loss modulus (*G*^″^) values in all frequency ranges, suggesting a viscoelastic solid character. As expected, emulsions with a higher fat content generally have a higher value of *G*′ than *G*^″^ [36]. In general, the *G*′ in full-fat emulsion is correlated with the strength of the attractive forces between the oil droplets [18]. The *G*′ of the reduced-fat salad dressing was enhanced with increasing inulin concentration (Figure 2(a)). The inulin dispersed in a continuous phase would have the ability to store the elastic energy of the reduced-fat emulsion under an applied force with different explanations. Inulin possesses hydrocolloid properties that can form a particulate gel-like network. The network structure could be stiffened by increasing inulin, resulting in a higher *G*′. In the literature, it was seen that when low-fat emulsions were supplemented with hydrocolloids, it typically demonstrated viscoelastic properties because the *G*′ was higher than *G*^″^, as reported in low-fat emulsions prepared with inulin and modified starch [18], extruded rice paste [20], pregelatinized potato starch [24], lentil flours [26], xanthan gum and tomato seed oil by-product [27], modified rice starch [28], 4*α*GTase-treated rice starch [29], *β*-glucan [31], pectin [37], nanofibrillated cellulose and guar gum [38], and oleaster flour [39].

### 3.3. Optical Microscopy

**Figure 3:**
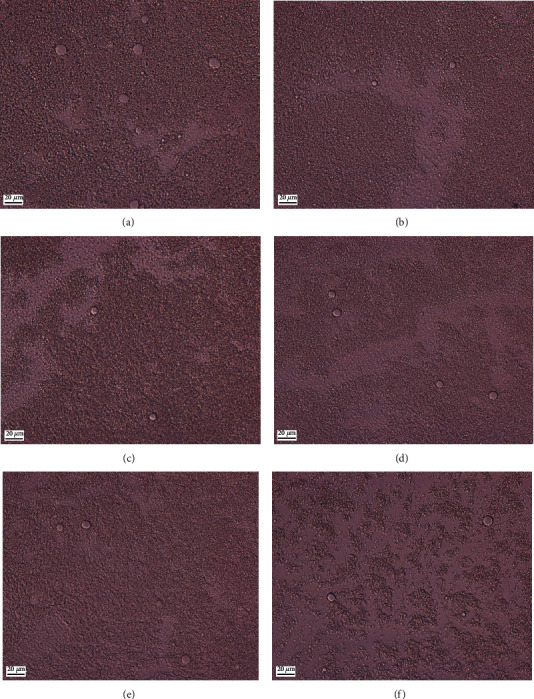
Optical micrographs of the (a) full-fat salad dressing and the reduced-fat salad dressing supplemented with inulin at (b) 10, (c) 12.5, (d) 15, (e) 17.5, and (f) 20%.

**Figure 4:**
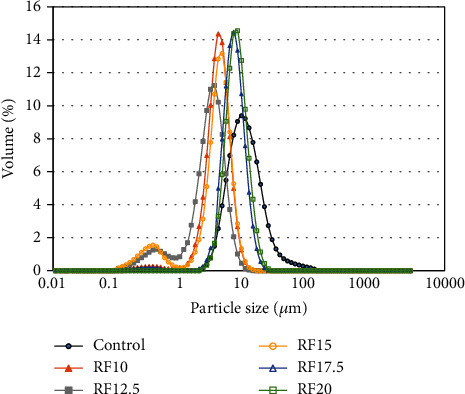
Particle size distribution of the full-fat salad dressing (control) and the reduced-fat salad dressing supplemented with inulin at 10 (RF10), 12.5 (RF12.5), 15 (RF15), 17.5 (RF17.5), and 20% (RF20).

Micrographs by optical microscope of full-fat and reduced-fat salad dressings are shown in Figure 3. The oil droplet sizes of all samples were smaller than 20 *μ*m. The oil droplet of the reduced-fat salad dressing was apparently larger with a higher inulin concentration (Figure 3). This result was in good agreement with the results obtained from instrumental analysis using a particle size analyzer (Figure 4). Generally, in high-oil-content emulsions, the greater extent of smaller oil droplet sizes tends to result in higher viscosity because the smaller droplets provide a larger interfacial area between the dispersed and continuous phases, leading to more interactions and a larger emulsion viscosity [22, 40]. However, in our findings, the reduced-fat emulsions with lower apparent viscosity (Figure 1) showed smaller oil droplets (Figure 3), explaining that a low flow resistance in an oil-in-water emulsion with a low concentration of inulin, as shown in Figure 1, can facilitate the breakdown of large fat droplets into smaller ones.

### 3.4. Particle Size Analysis

The particle size distribution of salad dressing emulsion in the full-fat and reduced-fat samples was mainly in the range of 1–20 *μ*m (Figure 4), as confirmed by microscope observation (Figure 3). The control emulsion had a peak particle size of 7.7 *μ*m. However, in the presence of inulin at concentrations of 10, 12.5, 15, 17.5, and 20%, the peak particle sizes were 3.4, 3.0, 3.9, 5.9, and 6.7 *μ*m, respectively (Figure 4). This result showed that the size of oil droplets increased when inulin was added, approaching the size of the control. The droplet size of these values was smaller than that reported in a commercial Italian salad dressing with droplet sizes ranging from 74 to 124 *μ*m [21], as well as a low-in-oil dressing emulsion containing orange pulp [4], pectin [25], pectin-weal gel [37], guar gum, and carboxyl methylcellulose [38], all with droplet sizes in the range of 100–1,000 *μ*m, 50–100 *μ*m, 80 *μ*m, 1-100 *μ*m, and 1-500 *μ*m, respectively. There has been a report that the emulsion droplet size in the range of 2–100 *μ*m generally provides a smooth appearance [22]. In preliminary data based on visual observations, the reduced-fat salad dressing made with inulin exhibited a significantly higher level of homogeneity, smoothness, and fine surface texture compared to the dressings made with xanthan gum, pectin, or guar gum (results not shown).

**Figure 5:**
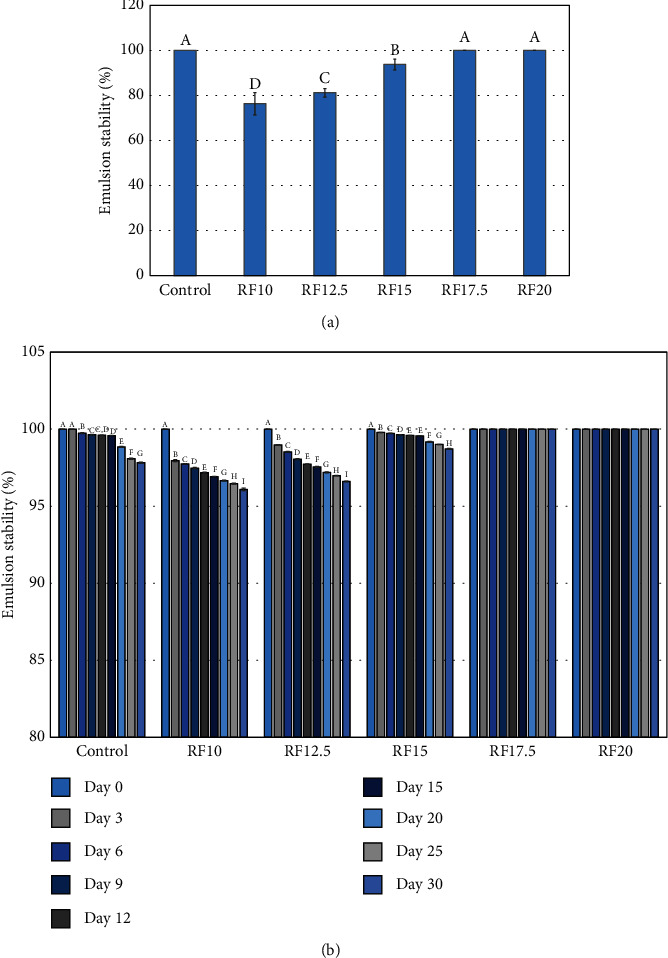
Emulsion stability of salad dressings. (a) Thermal stress tests: emulsion stability of full-fat (control) and reduced-fat salad dressings supplemented with inulin at 10 (RF10), 12.5 (RF12.5), 15 (RF15), 17.5 (RF17.5), and 20% (RF20) after exposure to thermal stress (80°C for 30 min) followed by centrifugation. Bars marked with different letters are significantly different at *p* ≤ 0.05. (b) Storage stability: emulsion stability of the same samples stored at 4°C for 30 days, measured using the centrifugation technique. Serum separation layers were not visually detected in any samples stored at 4°C before centrifugation. Bars marked with different letters within the same sample over the storage period represent statistically significant differences at *p* ≤ 0.05.

The surface-volume mean diameter (*D*[3, 2]) and the weight-average diameter (*D*[4, 3]), described as the average surface area and the average volume of the particles in the emulsion, respectively, were important factors in determining the emulsion stability. The particle sizes of droplets in full-fat and reduced-fat emulsions are shown in Table 3. The *D*[3, 2] and *D*[4, 3] values of the full-fat samples were 38.08 ± 5.45 *μ*m and 58.67 ± 7.52 *μ*m, respectively, which were larger than those of the reduced-fat samples (Table 3). If the values of *D*[3, 2] or *D*[4, 3] are smaller, it generally indicates a smaller average particle size in the emulsion, resulting in a more stable emulsion. However, the presence of inulin at 10 and 12.5% resulted in lower *D*[3, 2] and *D*[4, 3] values compared to the other samples, indicating less stability of the emulsion, as shown in Figures 5(a) and 5(b). This result indicated that the higher proportion of smaller droplets in the emulsion, as implied by higher emulsion stability, was an appropriate factor for full-fat emulsion, whereas in reduced-fat emulsion supplemented with inulin, viscosity would be a crucial factor for emulsion stability.

### 3.5. Emulsion Stability

The emulsion stability under thermal stress of the salad dressing samples is shown in Figure 5(a). The emulsion stability of salad dressing is one of the most important quality parameters, as it is prone to phase separation during storage. Generally, phase separation in an oil-in-water emulsion occurs because the droplets tend to merge with each other when they collide. The droplets dispersed in an aqueous phase are thermodynamically unstable due to the unfavorable contact between the oil and water molecules [41]. As expected, the reduced-fat salad dressing with 10 and 12.5% inulin showed relatively low emulsion stability (Figure 5(a)), which corresponded well with those lower viscosities (Figure 1). This result indicated that the addition of inulin at a low concentration (<15%) was not enough to achieve the desired viscosity in the continuous phase and prevent the movement and merging of oil droplets. The complete stabilization of the reduced-fat emulsions prepared with 17.5 and 20% inulin was observed to be the same as with the control (Figure 5(a)). The thermal stress test identified a salad dressing exhibiting good emulsion stability, as evidenced by minimal separation in Figure 5(a). However, while these tests indicate the potential for a longer shelf life due to the ability to withstand thermal stress during storage and transportation, they may not necessarily reflect the true emulsion stability over time. Therefore, the storage stability of the salad dressing samples at 4°C was investigated.  Figure 5(b) illustrates the emulsion stability, as determined by centrifugation, of the salad dressing stored at 4°C for 30 days. To emphasize changes in emulsion stability during storage, the *Y*-axis of Figure 5(b) starts at 80% emulsion stability. No visually detectable serum separation was observed in any samples stored at 4°C before centrifugation. However, centrifugation (4,000 × *g*, 15 min) revealed a cream phase, as shown in Figure 5(b). The serum separation layer of the reduced-fat salad dressings containing 10 and 12.5% inulin was initially observed on day 3 and gradually increased during storage (*p* ≤ 0.05) (Figure 5(b)). These two samples had significantly lower stability than the others during storage (*p* ≤ 0.05), which corresponded well with the results of the thermal stress test (Figure 5(a)). Our findings indicated that the reduced-fat salad dressings containing ≥15% inulin showed high emulsion stability at 4°C, similar to the control, even after 30 days of storage. When the amount of oil in an oil-in-water emulsion is reduced, the emulsion becomes unstable and requires the addition of hydrocolloids. There have been several reports that the addition of hydrocolloids in an oil-in-water emulsion improved the physical stability, as reported in several previous studies [18, 26–29, 31, 38, 39]. In fact, the inulin structure, consisting of free hydroxyl groups, would be capable of interacting with water molecules. The hydrated inulin as an individual particle form can interact with each other, leading to physical barriers that hinder the flow of the continuous phase in the emulsion. Therefore, higher inulin concentrations contributed to the better stability of the emulsion.

### 3.6. Sensory Evaluation

Sensory evaluation scores of the full-fat and reduced-fat salad dressings are shown in Table 4. The texture-liking score for the reduced-fat salad dressings increased as inulin concentrations increased to 17.5% (*p* ≤ 0.05) but decreased in the sample containing 20% inulin (*p* ≤ 0.05). The correlation between the texture-liking score and viscosity results (Figure 1(a)) appeared consistent. It is likely that the panelists were able to detect differences in texture among the samples that exhibited differences in viscosity. Moreover, it has been reported that inulin improved mouthfeel in foods [35], possibly contributing a texture-liking score on the hedonic scale (Table 4). Indeed, increasing concentrations of inulin led to higher liking scores for appearance, color, aroma, taste, and overall acceptability in the reduced-fat salad dressing (Table 4). Inulin generally contributes to a smooth, creamy texture and glossy appearance, making food products more visually appealing and enhancing color [42]. These results suggested that inulin not only affected texture but also influenced other organoleptic properties. Our findings revealed that there were no significant differences in appearance, color, aroma, texture, taste, and overall acceptability scores between full-fat and reduced-fat salad dressings with 15 and 17.5% inulin (*p* > 0.05). This indicated the potential use of inulin as a fat replacer in salad dressings without compromising sensory satisfaction.

## 4. Conclusion

This study demonstrated that inulin could be used to formulate reduced-fat salad dressing. The flow characteristics of all samples were non-Newtonian with shear-thinning behavior that was well described by the power law model, whose *n* and *K* parameters were estimated by fitting an exponential function. The reduced-fat salad dressing with 10–20% inulin showed elastic properties, as indicated by the higher *G*′ value compared to *G*^″^. The addition of inulin at higher levels (17.5 and 20%) in the reduced-fat sample resulted in a peak-size distribution of oil droplets that was comparable to that of full-fat salad dressing. The reduced-fat salad dressing supplemented with ≥15% inulin exhibited emulsion stability over 30 days of storage at 4°C compared to its full-fat counterpart (*p* ≤ 0.05). Sensory analyses of the reduced-fat salad dressings containing 15 and 17.5% inulin showed no significant difference compared to the control sample (*p* > 0.05). According to the results, the reduced-fat salad dressing containing high inulin content (≥15%) can be introduced as a fat replacer due to its similar rheological properties, sensory analyses, and emulsion stability compared to its full-fat counterpart.

## Figures and Tables

**Table 1:** Formulation (%, *w*/*w*) of full-fat and reduced fat-salad dressings.

Ingredient	Full-fat salad dressing (control)	Reduced-fat salad dressing with inulin supplementation				
		10% (RF10)	12.5% (RF12.5)	15% (RF15)	17.5% (RF17.5)	20% (RF20)
Vinegar	14	14	14	14	14	14
Sugar	18	18	18	18	18	18
Salt	2	2	2	2	2	2
Egg yolk	12	12	12	12	12	12
Soybean oil	54	27	27	27	27	27
Water	—	17	14.5	12	9.5	7
Inulin	—	10	12.5	15	17.5	20

**Table 2:** Rheological parameter of full-fat and reduced fat-salad dressings.

Parameter	Full-fat salad dressing (control)	Reduced-fat salad dressing with inulin supplementation				
		10% (RF10)	12.5% (RF12.5)	15% (RF15)	17.5% (RF17.5)	20% (RF20)
*K* (Pa·s^n^)	87.77 ± 0.53^d^	35.03 ± 0.09^e^	87.05 ± 5.18^d^	169.07 ± 11.06^c^	203.50 ± 2.29^b^	257.31 ± 11.23^a^
*n*	0.303 ± 0.001^a^	0.190 ± 0.003^b^	0.145 ± 0.001^c,d^	0.093 ± 0.017^e^	0.116 ± 0.002^d,e^	0.152 ± 0.025^c^
*R* ^2^	0.959	0.964	0.932	0.868	0.886	0.887

Different superscripts in each row indicate significant differences at *p* ≤ 0.05.

**Table 3:** The surface-volume mean diameter *D*[3, 2] and the weight-average diameter *D*[4, 3] of full-fat and reduced fat-salad dressing.

Particle size	Full-fat salad dressing (control)	Reduced-fat salad dressing with inulin supplementation				
		10% (RF10)	12.5% (RF12.5)	15% (RF15)	17.5% (RF17.5)	20% (RF20)
*D*[3, 2] (*μ*m)	38.08 ± 5.45^a^	4.24 ± 0.05^e^	4.11 ± 0.55^f^	4.85 ± 0.46^d^	7.91 ± 1.08^b,c^	8.94 ± 0.84^b^
*D*[4, 3] (*μ*m)	58.67 ± 7.52^a^	4.76 ± 0.01^b^	4.66 ± 0.58^b^	5.43 ± 0.51^b^	8.87 ± 1.14^b^	10.06 ± 0.96^b^

Different superscripts in each row indicate significant differences at *p* ≤ 0.05.

**Table 4:** Sensory evaluation of the full-fat and reduced fat-salad dressings.

Sample^∗^	Appearance	Color	Aroma	Texture	Taste	Overall acceptability
Control	7.23 ± 1.20^a^	7.37 ± 1.21^a^	6.14 ± 1.40^a,b^	7.13 ± 1.31^a^	7.01 ± 1.24^a,b^	7.13 ± 1.12^a^
RF10	6.10 ± 1.61^c^	6.55 ± 1.56^b^	5.87 ± 1.48^b^	5.72 ± 1.64^c^	6.44 ± 1.57^c^	6.24 ± 1.36^c^
RF12.5	6.61 ± 1.46^b^	6.88 ± 1.41^b^	6.26 ± 1.43^a,b^	6.37 ± 1.55^b^	6.69 ± 1.52^b,c^	6.72 ± 1.27^b^
RF15	7.11 ± 1.28^a^	7.46 ± 0.95^a^	6.36 ± 1.45^a^	7.09 ± 1.27^a^	6.99 ± 1.33^a,b^	7.00 ± 1.18^a,b^
RF17.5	7.33 ± 1.18^a^	7.63 ± 1.07^a^	6.28 ± 1.38^a,b^	7.01 ± 1.18^a^	7.25 ± 1.34^a^	7.28 ± 0.98^a^
RF20	6.98 ± 1.31^a^	7.26 ± 1.39^a^	6.37 ± 1.50^a^	6.21 ± 1.53^b^	7.02 ± 1.35^a,b^	6.98 ± 1.16^a,b^

^∗^Control represents the full-fat salad dressing; RF10, RF12.5, RF15, RF17.5, and RF20 represent reduced-fat salad dressings containing inulin at concentrations of 10, 12.5, 15, 17.5, and 20%, respectively, used as a fat replacer with a 50% oil substitution level. Different superscripts within each column indicate significant differences at *p* ≤ 0.05.

## Data Availability

The data analyzed during this study are all included in the main manuscript.
